# Dynamic serum albumin and outcome of peritoneal dialysis patients: A retrospective study in China

**DOI:** 10.3389/fmed.2022.917603

**Published:** 2022-08-02

**Authors:** Panai Song, Dong Yang, Jine Li, Ning Zhuo, Xiao Fu, Lei Zhang, Hongqing Zhang, Hong Liu, Lin Sun, Yinghong Liu

**Affiliations:** Department of Nephrology, The Second Xiangya Hospital of Central South University, Hunan Key Laboratory of Kidney Disease and Blood Purification, Changsha, China

**Keywords:** time-averaged albumin, serum albumin reach rate, peritoneal dialysis, all-cause mortality, cardiovascular mortality

## Abstract

**Introduction:**

Serum albumin levels at a single time point have been shown to predict mortality in peritoneal dialysis (PD) patients. However, we believe that the dynamic change in albumin after PD may be more significant. In this study, we investigated the relationship between dynamic serum albumin and the clinical outcome of patients undergoing continuous ambulatory peritoneal dialysis (CAPD).

**Methods:**

The participants in this study enrolled 586 patients who underwent CAPD at the peritoneal dialysis center of Second Xiangya Hospital in China. We retrospectively reviewed medical records from January 1, 2010, to December 31, 2019. Baseline serum albumin (Alb), time-averaged albumin level (TA-ALB) and serum albumin reach rate (SR: defined as the percentage of serum albumin measurements that reached ≥ 35 g/L) were applied as the predictor variables. All-cause mortality and cardiovascular mortality were used as the outcome variables. Hazard function of all-cause mortality and cardiovascular mortality in the study participants were examined by using Cox proportional hazard regression models.

**Results:**

Age (HR = 1.03, 95% CI 1.00–1.05), cardiovascular disease (HR = 1.80, 95% CI 1.07–3.03) and TA-ALB (HR = 0.92, 95% CI 0.85–0.99) were independent risk factors for all-cause mortality in PD patients. Patients with TA-ALB of <33 g/L (HR = 2.33, 95% CI 1.17–4.62) exhibited a higher risk for all-cause mortality than those with TA-ALB ≥ 36 g/L. Stratified SR showed a similar trend. Patients with a <25% SR exhibited a significantly increased risk for all-cause mortality (HR = 2.72, 95% CI, 1.24–5.96) by fully adjusted analysis. However, neither TA-ALB nor SR were associated with the risk of cardiovascular mortality after adjusted analysis.

**Conclusion:**

This study demonstrated that age, cardiovascular disease, and TA-ALB were independent risk factors for all-cause mortality in PD patients. TA-ALB and SR can better predict the prognosis of PD patients than baseline Alb. Dynamic changes in Alb are more clinically significant than baseline Alb in predicting mortality risk.

## Introduction

Peritoneal dialysis (PD) is an important kidney replacement therapy. It has been reported that PD protected residual renal functions better and had other advantages in many studies (1). Therefore the number of PD patients is increasing year by year. It is estimated that there are more than 272,000 PD patients worldwide, accounting for around 11% of the dialysis population (2). Despite the technology and treatments for PD have improved over the years, many PD patients still develop metabolic disorders, such as hypokalemia and hypoproteinemia, leading to an increased risk of mortality (3).

Protein-energy wasting (PEW) is a common metabolic disorder in PD patients (4) 4, and serum albumin (Alb) is an important index to evaluate PEW. The causes of hypoalbuminemia in PD patients are complex, including protein loss during peritoneal dialysis, inflammation, decreased protein intake, chronic acidosis, and psychosocial factors (5). Clear evidence has shown that hypoalbuminemia was closely related to all-cause mortality and cardiovascular(CV) mortality in PD patients (6, 7). However, Olga et al. (8) have reported that baseline peritoneal loss and albumin clearance was not a determinant of survival. Another study (9) has demonstrated that albumin trajectories after PD was better than initial serum albumin level in predicting mortality risk. Therefore, it may be more meaningful to analyze the association of dynamic serum albumin changes with the long-term survival of dialysis patients.

The aim of this cohort study was to investigate whether serum albumin changes over time are predictors of changes in survival in patients undergoing continuous ambulatory peritoneal dialysis (CAPD). We measured 3 predictor variables, target baseline albumin (Alb), time-averaged albumin (TA-ALB) levels, and albumin standard reach rates (SR), to predict all-cause mortality and CV mortality.

## Materials and methods

### Study population

**FIGURE 1:**
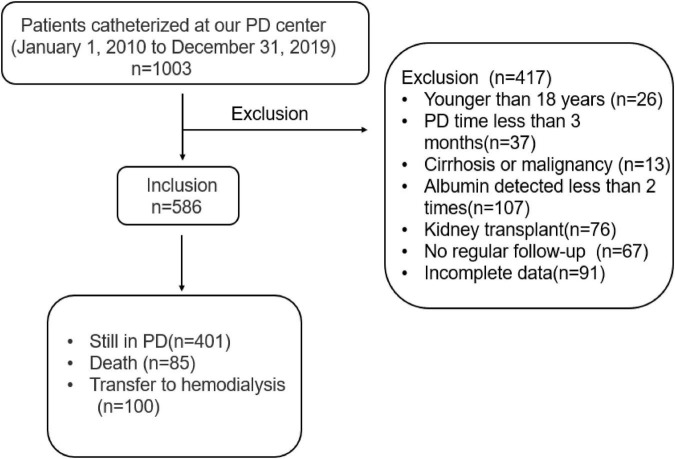
Flow chart for the study participants enrollment and outcomes.

In this study, as shown in Figure 1, a total of 1,003 patients catheterized and received CAPD treatment from January 1, 2010 to December 31, 2019 at PD center of Second Xiangya Hospital, Central South University in China were reviewed. We excluded 403 patients because of incomplete demographic and laboratory data, and 586 patients were considered eligible for the survival analysis. The exclusion criteria were as follows: (1) age < 18 years, (2) incomplete clinical information, (3) duration of PD was less than 3 months, (4) cirrhosis or malignancy, (5) serum albumin was measured less than 2 times, (6) kidney transplantation, and (7) no regular follow-up was performed 1–3 months after catheterization. Approval of the study by the research ethics committee of Central South University and informed consents were provided prior to their inclusion in the study.

### Stratified serum albumin levels

We used Alb, TA-ALB level, and SR as the predictor variables. All-cause mortality and CV mortality were used as the outcome variables. Two serum albumin stratifications were used for analyses.

TA-ALB: according to the trapezoidal area method (10), the area under the curve is formed by albumin values divided by the follow-up time. The formula is as follows: where B is serum albumin values, T is the follow-up time (the month after catheterization), and N is the frequency of follow-up (*n* ≥ 2).


$$T\,A-A\,L\,B=\frac{\begin{array}{c} \left({\text{B}}_{1}+{\text{B}}_{2}\right)\,\left({\text{T}}_{2}-{\text{T}}_{1}\right)+\left({\text{B}}_{2}+{\text{B}}_{3}\right)\,\left({\text{T}}_{3}-{\text{T}}_{2}\right)\\ +\ldots .+\left({\text{B}}_{\text{n}-1}+{\text{B}}_{\text{n}}\right)\,\left({\text{T}}_{\text{n}}-{\text{T}}_{\text{n}-1}\right) \end{array}}{2\,\left({\text{T}}_{\text{n}}-{\text{T}}_{1}\right)}$$

And according to the rule of tertiles, TA-ALB was divided into three groups (A1 ≤ 33 g/L, A2:33 g/L-36 g/L, A3 ≥ 36 g/L).

SR: SR is the percentage of albumin measurements reaching ≥ 35 g/L. We stratified the albumin reach rate into 4 groups depending on the serum albumin levels that reached 35 g/L: S1: SR < 25%, S2: 25% ≤ SR < 50%, S3: 50% ≤ SR < 75%, S4 ≥ 75%, 25% albumin reach rate indicated that a quarter of serum albumin measurements had reached 35 g/L, 50% indicated that half of serum albumin measurements had reached 35 g/L, and 75% indicated that three-quarters of serum albumin measurements had reached 35 g/L.

### Outcome measures

All-cause mortality and cardiovascular mortality were recorded as endpoint events. Cardiovascular mortality was defined as cardiac arrest, myocardial infarction, arrhythmia, heart failure, cerebrovascular accident, and other heart-related diseases. All-cause mortality is defined as death from any cause.

### Statistical analysis

Results were expressed as count and percentage or median and interquartile range, as appropriate. Categorical variables were compared using the chi-squared test and Fisher’s exact test. Independent risk factors of all-cause mortality in PD patients were analyzed by univariate and multivariate Cox regression analysis. The correlations between Alb, TA-ALB, SR and all-cause mortality, and cardiovascular mortality were evaluated by Cox proportional hazards model analysis. Survival curves were generated using the Kaplan–Meier method. SPSS 26.0 was used for all statistical analyses, and the *P*-value of <0.05 was considered statistically significant.

## Results

### Clinical characteristics of different time-averaged albumin level groups

A total of 586 patients were analyzed. During the 10 years, 203 patients had higher albumin levels (A3: TA-ALB > 36 g/l), whereas 196 patients had low albumin levels (A1: TA-ALB < 33 g/l) and 187 patients had intermediate albumin levels (A2: 33 g/l < TA-ALB < 36 g/l). Patients who were older, male, diabetic, and had high use rate of diuretics had lower serum albumin. Moreover, patients with low albumin had lower rates of glomerular disease, lower residual renal function, and higher rates of ACEI use, cardiovascular disease, all-cause mortality, and cardiovascular mortality. Data of blood parameters showed that patients with lower albumin exhibited significantly higher C-reactive protein(CRP), lower Hb, albumin uric acid(UA), calcium(Ca), phosphorus(P), and magnesium(Mg) values than those with higher albumin. However, the ratio of monocytes to lymphocytes was significantly higher in patients with lower albumin (Table 1).

**TABLE 1:** Baseline characteristics and clinical features of the study population according to different TA-ALB groups.

	A1 (*N* = 196)	A2 (*N* = 187)	A3 (*N* = 203)	*p*
	TA-ALB < 33 g/L	TA-ALB 33–36 g/L	TA-ALB ≥ 36 g/L	
Age(year)	53.8 ± 14.0	50.1 ± 12.5*	45.4 ± 12.1*	<0.001
Gender(male)	113 (57.7%)	81 (43.3%)*	88 (43.3%)*	0.005
**Primary disease**				
Glomerulonephritis	99 (50.5%)	102 (54.5%)	146 (71.9%)*Δ	<0.001
Diabetic nephropathy	32 (16.3%)	19 (10.2%)	8 (3.94%)*Δ	<0.001
Hypertensive nephropathy	36 (18.4%)	26 (13.9%)	22 (10.8%)	0.098
**Medication**				
ACEI	73 (37.2%)	55 (29.4%)	48 (23.6%)*	0.012
β-blockers	91 (46.4%)	79 (42.2%)	85 (41.9%)	0.600
CCB	173 (88.3%)	170 (90.9%)	168 (83.2%)	0.064
Diuretics	45 (23.0%)	24 (12.8%)*	25 (12.3%)*	0.005
**Complications**				
Cardiovascular disease	43 (21.9%)	25 (13.4%)	15 (7.39%)*	<0.001
Diabetes mellitus	47 (24.0%)	23 (12.3%)*	14 (6.90%)*	<0.001
**Outcomes**				
Peritonitis	49 (25.0%)	44 (23.5%)	49 (24.1%)	0.944
All-cause mortality	44 (22.4%)	26 (13.9%)	15 (7.39%)*	<0.001
Cardiovascular death	28 (14.3%)	16 (8.56%)	7 (3.45%)*	0.001
Hemodialysis	39 (19.9%)	31 (16.6%)	30 (14.8%)	0.388
**Laboratory findings**				
White blood cell (10^9/L)	6.0(4.8,7.4)	6.0(5.0,7.5)	6.1(5.1,7.4)	0.748
Hemoglobin (g/L)	95 **±** 19	98 **±** 17	104 **±** 18*Δ	<0.001
Platelets (10^9/L)	196(154,243)	191(151,246)	195(162,250)	0.771
Neutrophils (10^9/L)	3.9(3.1,5.3)	4.0(3.3,5.3)	4.1(3.3,5.1)	0.658
M/L	0.25(0.19,0.34)	0.23(0.18,0.30)*	0.21(0.17,0.28)*	0.001
Urea nitrogen (mmol/L)	19.0(15.2,23.3)	19.7(16.6,26.3)	20.0(16.2,25.3)	0.064
Creatinine (umol/L)	730(567,974)	777(653,986)	749(624,974)	0.186
Uric acid (umol/L)	411 **±** 95.8	442 **±** 99.9*	455 **±** 103*	<0.001
Calcium (mmol/L)	2.00(1.86,2.09)	2.10(1.95,2.19)*	2.11(1.97,2.25)*	<0.001
Phosphorus (mmol/L)	1.49(1.28,1.83)	1.60(1.37,1.92)*	1.60(1.34,1.94)*	0.029
Sodium(mmol/L)	141(139,143)	140(139,142)	141(139,142)	0.518
Potassium (mmol/L)	4.09(3.60,4.57)	4.05(3.67,4.62)	4.00(3.60,4.52)	0.409
Magnesium (mmol/L)	0.89(0.79,0.98)	0.95(0.88,1.05)*	0.99(0.88,1.08)*	<0.001
Albumin (g/L)	30.9 **±** 4.04	35.1 **±** 3.38*	38.0 **±** 3.49*	<0.001
Total protein (g/L)	58.5 **±** 6.76	64.0 **±** 7.20*	66.7 **±** 6.97*	<0.001
CRP (mg/dL)	3.25(1.41,8.15)	2.16(0.92,6.28) *	1.81(0.68,3.94) *Δ	<0.001
iPTH (mg/dL)	26.9(17.5,38.6)	29.1(15.6,43.4)	28.7(16.8,45.1)	0.824
BMI (kg/m^2^)	21.6(19.6,23.8)	21.8(19.8,24.0)	21.3(19.1,23.5)	0.651
Total Kt/v	1.8(1.5,2.2)	1.8(1.6,2.3)	1.9(1.5,2.4)	0.095
Total Ccr	59.9(47.3,80.2)	60.6(49.2,77.7)	64.1(49.8,77.2)	0.712
eGFR (mL/min/1.73 m^2^)	3.1(1.5,5.1)	3.1(1.7,4.9)	3.7(2.3,5.4)*Δ	0.015

BMI, body mass index; Total Kt/V, total urea clearance index; Total Ccr, total creatinine clearance rate; eGFR, glomerular filtration rate; ACEI, angiotensin enzyme inhibitor; CCB, calcium channel blocker; M/L, monocytes/lymphocytes; CRP, C-reactive protein; iPTH, ionization parathyroid hormone; *, compared to group A1, P < 0.05; Δ, compared to group A2, P < 0.05.

### Independent risk factors analysis of all-cause mortality in peritoneal dialysis patients

In univariate Cox regression analysis, age, diabetes, UA, Alb, TA-ALB, serum potassium, and cardiovascular disease (CVD) were chosen for adjustment for multivariate Cox proportional-hazards model analysis. We found that age(HR = 1.03, 95%CI 1.00–1.05, *p* = 0.012), CVD(HR = 1.80, 95%CI 1.07–3.02, *p* = 0.028), and TA-ALB(HR = 0.92, 95%CI 0.85–0.99, *p* = 0.028)were independent risk factors for all-cause mortality (Table 2).

**TABLE 2:** Independent risk factors analysis of all-cause mortality in PD patients.

Variable	Univariate Cox regression analysis			Multivariate Cox regression analysis		
	HR	95%CI	*p*	HR	95%CI	*p*
Age (year)	1.06	1.04–1.07	<0.001	1.03	1.00–1.05	0.012
Primary disease						
Glomerulonephritis	0.58	0.31–1.11	0.101			
Diabetic nephropathy	2.07	1.00–4.26	0.049			
Hypertensive nephropathy	1.82	0.92–3.60	0.085			
Other	1					
Uric acid (umol/L)	0.99	0.99–1.00	0.005			
Albumin (g/L)	0.93	0.89–0.97	0.002			
TA-ALB(g/L) Potassium(mmol/L)	0.87 0.68	0.83–0.92 0.49–0.95	<0.001 0.022	0.92	0.85–0.99	0.028
Cardiovascular disease	2.44	1.55–3.85	<0.001	1.80	1.07–3.03	0.028

TA-ALB: Time averaged albumin; HR: hazard ratio, CI: confidence interval.

### Correlations between albumin, time-averaged albumin levels, standard reach rates, and all-cause mortality and cardiovascular mortality

Based on Cox regression analysis, Table 3 shows the association between different albumin stratification levels and all-cause mortality and cardiovascular mortality. Baseline Alb, TA-ALB, and SR were inversely associated with the risk of all-cause mortality and cardiovascular mortality. Adjusted analysis showed that the risk of all-cause mortality in the participants increased parallel to low serum albumin. Participants with TA-ALB levels < 33 g/L exhibited a higher risk for all-cause mortality as compared with those with the reference level (≥ 36 g/L) (HR = 2.33, 95% CI 1.17–4.62). Stratified SR showed a similar trend. Participants with a < 25% SR had the highest statistically significant risk for all-cause mortality (HR = 2.72, 95% CI, 1.24–5.96). However, neither TA-ALB nor SR were associated with the risk of cardiovascular mortality after fully adjusted analysis.

**TABLE 3:** Correlations between Alb, TA-ALB, SR, and all-cause mortality, cardiovascular mortality in the study population.

	Model 1		Model 2		Model 3	
	HR(95%CI)	*P*	HR(95%CI)	*P*	HR(95%CI)	*P*
**All-cause mortality**						
Alb(g/L)	0.93(0.89,0.97)	0.002	0.99(0.93,1.05)	0.700	0.99(0.93,1.06)	0.830
TA-ALB(g/L)	0.87(0.83,0.92)	<0.001	0.94(0.88,1.00)	0.046	0.93(0.87,1.00)	0.043
A3	Reference		Reference		Reference	
A2	2.06(1.09,3.89)	0.026	1.52(0.78,2.94)	0.217	1.51(0.77,2.94)	0.223
A1	3.79(2.10,6.82)	<0.001	2.20(1.13,4.30)	0.020	2.29(1.15,4.58)	0.019
SR						
S4	Reference		Reference		Reference	
S3	2.47(1.16,5.26)	0.019	2.07(0.96,4.48)	0.064	1.91(0.88,4.18)	0.103
S2	2.88(1.30,6.42)	0.009	2.17(0.94,4.97)	0.068	2.25(0.97,5.25)	0.059
S1	4.64(2.31,9.34)	<0.001	2.78(1.29,6.00)	0.009	2.72(1.24,5.96)	0.013
**Cardiovascular mortality**						
Alb(g/L)	0.91(0.86,0.97)	0.001	0.97(0.90,1.06)	0.508	0.97(0.89,1.06)	0.494
TA-ALB(g/L)	0.87(0.81,0.93)	<0.001	0.97(0.89,1.06)	0.465	0.97(0.88,1.06)	0.480
A3	Reference		Reference		Reference	
A2	2.71(1.11,6.59)	0.028	1.80(0.71,4.56)	0.217	1.76(0.68,4.55)	0.243
A1	5.14(2.24,11.80)	<0.001	2.38(0.93,6.08)	0.071	2.35(0.89,6.21)	0.086
SR						
S4	Reference		Reference		Reference	
S3	1.92(0.70,5.28)	0.208	1.50(0.53,4.23)	0.448	1.43(0.50,4.08)	0.508
S2	2.56(0.89,7.39)	0.081	1.78(0.58,5.41)	0.311	1.62(0.51,5.15)	0.416
S1	5.30(2.18,12.89)	<0.001	2.56(0.96,6.84)	0.062	2.56(0.92,7.07)	0.071

Univariate and multivariate Cox proportional hazard regression models. Model 1: unadjusted. Model 2: adjusted for demographic variables including age, sex, diabetes mellitus and laboratory variables including White blood cell, hemoglobin, platelet, M/L, neutrophil, blood calcium, blood phosphorus, blood potassium, blood sodium, blood magnesium, urea nitrogen, creatinine, uric acid, C-reactive protein, iPTH. Model 3: adjusted for all demographic and laboratory variables including Total KT/V, Total CCR, eGFR, ACEI, β-blockers, CCB, diuretics and peritonitis. HR, hazard ratio, CI, confidence interval.

### Cumulative survival

**FIGURE 2:**
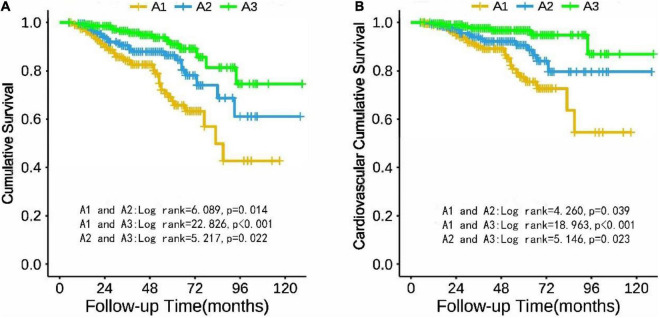
Cumulative survival of PD patients in TA-ALB groups (A1, A2, and A3) with all-cause mortality **(A)** and cardiovascular mortality **(B)** as end points.

Taking all-cause mortality of the CAPD patients as the endpoint, cumulative survival was analyzed. The 1-, 3-, and 5-year survival rates of the A1 group were 94.1, 83.6, and 67.4%; A2 group were 99.1, 89.2, and 85.1%; A3 group were 99.0, 95.8, and 90.8%, respectively. Figure 2A describes the cumulative survival of three groups of TA-ALB using the Kaplan-Meier analysis. The cumulative survival of each group was significantly different. Taking CV mortality as the endpoint, the 1-, 3-, and 5-year survival rates of the A1 group were 98.7, 87.4, and 76.1%; A2 group were 99.1, 93.5, and 89.4%; A3 group were 99.5, 97.2, and 95.2%, respectively. Kaplan-Meier analysis also showed a significant difference in cumulative survival among the three groups (Figure 2B).

## Discussion

This retrospective cohort study examined the association of baseline Alb, TA-ALB, and SR with all-cause and cardiovascular mortality in PD patients. The results showed that TA-ALB is an independent risk factor for all-cause mortality in PD patients, a decrease in TA-ALB and a low SR were associated with an increased risk of all-cause mortality in PD patients. Dynamic changes in Alb can better predict the risk of all-cause mortality in PD patients.

Age (11), gender (12), and comorbidities (13) have been shown to influence the prognosis of dialysis patients. This study found that age, CVD, and TA-ALB were independent risk factors for all-cause death in PD patients. We also found that patients with hypoalbuminemia were older and had higher rates of diuretic use, male and DM. The weakened immunity of elderly patients and the susceptibility to infection caused by improper operation, higher peritoneal transport and peritoneal permeability in DM patients than non-diabetic patients (14), and more leakage of Alb may explain the above findings. It has been reported that female patients receiving dialysis have an increased risk of infection (15), which is more likely to lead to malnutrition. However, our study found that the proportion of males in the low serum albumin group was higher, which may be related to the influence of regional differences.

The decline of RRF is closely related to inflammation, malnutrition, and death in PD patients (16, 17). Alb can reflect inflammation and malnutrition, which explains the higher CRP, lower eGFR and lower Hb, UA, Ca, P, Mg in the low albumin group. In this study, the use of diuretics and ACEIs was higher in the A1 group, which may be related to the increased edema and increased volume load caused by low Alb, leading to increased blood pressure. M/L are novel indicators of baseline inflammatory response (18). We speculated that the higher M/L in A1 group might be related to the inflammatory state.

PD patients’ prognosis has always been a concern of clinicians. Initial or single-time point serum albumin is commonly considered to be important in predicting the prognosis of dialysis patients (19–21). Sharma et al. (20) proposed that Alb at the start of PD was a better predictor of mortality than Alb after PD initiation. However, in recent years, some studies considered time-varying changes in serum albumin levels in examining the relationship of serum albumin levels with mortality in dialysis patients (22, 23). Wang et al. (9) demonstrated that albumin trajectories after PD was better than initial serum albumin level in predicting mortality risk. In this study, we assumed the dynamic change and trend of albumin after PD was essential and investigated the relationship of TA-ALB and SR with mortality in PD patients. We found that PD patients with lower TA-ALB and lower SR demonstrated higher all-cause mortality. The results demonstrated the long-term effect of serum albumin levels on the mortality risk of PD patients and showed that a sustained serum albumin level is crucial for maintaining survival benefits in PD patients.

Studies have shown that Alb is also a predictor of cardiovascular death in PD patients. One study suggested that after adjusting for other risk factors, the risk of cardiovascular death increased by more than 10-fold in Alb < 35 g/L patients (24). Another study showed that when Alb < 30 g/L, the risk of cardiovascular mortality was increased in both PD and HD patients (25). Our study showed that cardiovascular mortality was increased when PD patients with TA-ALB < 33 g/L. After adjusting for related factors, the risk of cardiovascular mortality remained higher in patients with low TA-ALB, although there was no statistically significant difference (*P* = 0.067), which may have been influenced by the small number of cardiovascular death endpoint events in this study.

Our study is rare in comparing the effects of baseline Alb, TA-ALB, and SR on all-cause mortality and CV mortality in PD patients. We find that TA-ALB and SR can better reflect the dynamic changes of Alb over time and predict the survival outcome of PD patients. Of course, there are still some limitations in this study. As a retrospective cohort study, we could not prove the causal relationship between TA-ALB, SR, and all-cause mortality, so more prospective studies are needed to confirm this in the future. Inflammation indicators such as CRP and IL-6 were not included in the model when we adjusted for confounding factors, which may impact the results. In addition, this study is a single-center study with a small sample size, and a multi-center randomized controlled study is needed to verify this conclusion.

## Conclusion

This study demonstrated that age, cardiovascular disease and TA-ALB were independent risk factors for all-cause mortality in PD patients. TA-ALB and SR can better predict the prognosis of PD patients than baseline Alb. Dynamic changes in Alb are more clinically significant than baseline Alb in predicting mortality risk. Increasing the albumin level over time can improve the prognosis of PD patients.

## Data availability statement

The original contributions presented in the study are included in the article/supplementary material, further inquiries can be directed to the corresponding author/s.

## Ethics statement

The studies involving human participants were reviewed and approved by the Ethics Committee of Second Xiangya Hospital. The patients/participants provided their written informed consent to participate in this study.

## Author contributions

PS collected the clinical data and drafted and revised the manuscript. JL, DY, and HZ collected the clinical data and searched the relative literatures. NZ searched the relative literatures, made analysis, and revised the English of the manuscript. XF, LZ, HL, and LS provided with the clinical assistance and contributed to the acquisition of these data. YL revised the manuscript and takes responsibility for the work. All authors have read and approved the final version.
